# Closing in on a new treatment for sleeping sickness

**DOI:** 10.7554/eLife.01042

**Published:** 2013-07-09

**Authors:** Emily R Derbyshire, Jon Clardy

**Affiliations:** 1 **Emily R Derbyshire** is in the Department of Biological Chemistry and Molecular Pharmacology, Harvard Medical School, Boston, United Statesemily_derbyshire@hms.harvard.edu; 2 **Jon Clardy** is an *eLife* reviewing editor, and is in the Department of Biological Chemistry and Molecular Pharmacology, Harvard Medical School, Boston, United Statesjon_clardy@hms.harvard.edu

**Keywords:** chemical biology, *T. brucei*, chemoproteomics, hypothemycin, protein kinase, Mouse, Other

## Abstract

A chemoproteomics approach has been employed to identify a kinase that could be used as a druggable target in efforts to develop new treatments for African sleeping sickness.

**Related research article** Nishino M, Choy JW, Gushwa NN, Oses-Prieto JA, Koupparis K, Burlingame AL, Renslo AR, McKerrow JH, Taunton J. 2013. Hypothemycin, a fungal natural product, identifies therapeutic targets in *Trypanosoma brucei*. *eLife* **2**:e00712. doi: 10.7554/eLife.00712

**Image** Hypothemycin could lead to new treatments for sleeping sickness

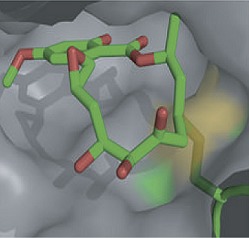

Chemists have created impressive collections of small molecules in recent years. The Molecular Libraries Program, for example, contains information on more than 300,000 different molecules, and PubChem lists chemical structures and the results of experimental investigations for some 5.5 million unique molecules. This immense amount of chemical information, combined with the availability of fully sequenced genomes for a growing number of hosts and pathogens, could in theory accelerate efforts to identify new drugs for the treatment of a variety of diseases. In practice, however, sampling the complete chemical space to discover molecules with therapeutic potential is inefficient. Instead, using a handful of carefully selected compounds that identify a limited set of targets may be a more fruitful means of uncovering candidate drugs.

African sleeping sickness, or trypanosomiasis, is a neglected tropical disease caused by the protozoan parasite *Trypanosoma brucei* (Kennedy, 2013). Untreated, the disease is often fatal, and the latest treatment, eflornithine, while still effective, is over 50 years old. The genomes of several strains of *T. brucei* have been sequenced, and together they contain genes that code for about 9000 proteins, including about 1700 that are specific to *T. brucei* (Berriman et al., 2005). Now, in *eLife*, Jack Taunton and colleagues at the University of California, San Francisco—including Mari Nishino, Jonathan Choy and Nathan Gushwa as joint first authors—report a chemoproteomics approach to focus their search for drug targets in a way that creates a clear path to new trypanosomiasis therapies (Nishino et al., 2013).

Chemoproteomics is a relatively recent entry in the -omics lexicon, and involves using chemical tools, often small molecules, to identify a group of functionally related proteins (Moellering and Cravatt, 2012). The UCSF team began by selecting protein kinases as their intended targets because inhibiting these enzymes in other systems is often lethal. As well as playing essential roles in the cell, kinases are druggable: that is, small molecules have been developed that can selectively inhibit these proteins in a variety of screening assays. More importantly, some of these small-molecule inhibitors have become effective drugs: Gleevec (imatinib), the tyrosine-kinase inhibitor used to treat chronic myelogenous leukemia and other cancers, is probably the best known.

The *T. brucei* genome encodes 182 protein kinases, most with poorly understood functions. The UCSF team focused on a subset of 21 *T. brucei* kinases that had a cysteine residue near their active sites (Schirmer et al., 2006). Most of these ‘CDXG kinases’, named after the motif found at their active sites, were expected to react with and be inhibited by a proven CDXG inhibitor called hypothemycin (Nair and Carey, 1980; Agatsuma et al., 1993) (Figure 1). Hypothemycin is a compound produced by several fungal species, and it can inhibit some CDXG kinases by reacting with a cysteine residue at the active site.

**Figure 1.**
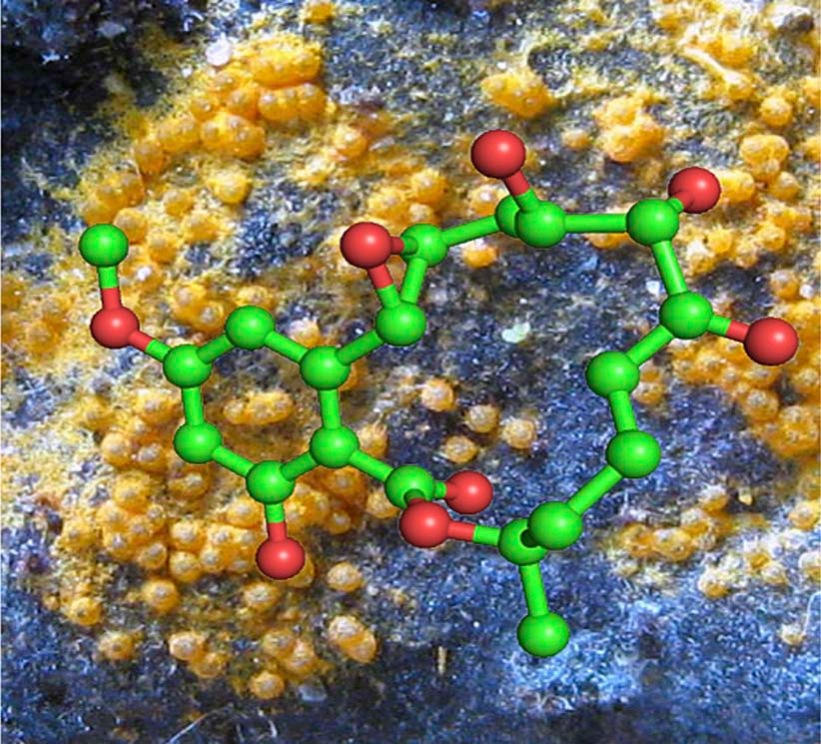
The kinase inhibitor hypothemycin has been used to identify a druggable kinase to combat African sleeping sickness. Here a ball-and-stick molecular model of hypothemycin is overlaid on an image of *Hypomyces subiculosus* (orange), the fungus from which it was originally isolated over thirty years ago.

PHOTOGRAPH: MASANORI KUTSUNA

The UCSF team first established that hypothemycin killed *T. brucei* at therapeutically significant (sub-µM) levels in vitro, and then found that it reduced the parasite load in vivo in a mouse model. However, higher levels of hypothemycin resulted in toxicity, which means that the dose cannot be increased to a level that would completely eliminate the parasites. CDXG kinases are conserved in mammals, and these results highlight the liability of kinase inhibitors that lack sufficient host/parasite selectivity. Significantly, however, while hypothemycin could kill parasites in a mammalian host, it was not established that it did so by inhibiting CDXG kinases, or if it did, which of the 21 such kinases in *T. brucei* was affected.

The team then employed an impressive array of experimental techniques in an effort to identify the kinase that was targeted by hypothemycin. They also chemically modified hypothemycin to make it easy to detect (without altering its biological activity). In a follow-up assay, this modified hypothemycin labeled 11 proteins, all of which were CDXG kinases. More quantitative studies reduced these 11 potential targets to two, and still further studies fingered a single kinase called *Tb*CLK1. An RNAi knockdown of *Tb*CLK1 independently showed that it was essential in *T. brucei.*

What did the chemoproteomics approach accomplish in this study? First, it allowed Taunton and colleagues to define the activity of a single molecule, hypothemycin, in a whole-organism assay that, by its very nature, includes all targets. Secondly, since hypothemycin had a known mechanism of action—covalently inhibiting a small class of kinases by reaction with a critical cysteine residue—the initial search for the target was greatly simplified. By narrowing down the original set of plausible suspects to *Tb*CLK1, the UCSF team illustrated the power of systematically applying several new technologies.

An important point, which Taunton and co-workers diplomatically mention only in passing, is that more inclusive assays to identify druggable *T. brucei* kinases failed to capture *Tb*CLK1 as a potential therapeutic target. This discrepancy likely results from the experimental differences between probing cell lysates with small molecules and probing whole organisms. So the hypothemycin-based chemoproteomics approach, with its early attention to mechanism and target structure, yielded a significant dividend.

Hypothemycin’s disadvantages as a therapy, including the fact that it inhibits several human kinases (Schirmer et al., 2006), will prevent it from being developed as a new drug to treat human African trypanosomiasis. But there is room for optimism, as large numbers of kinase inhibitors already exist and may be repurposed to inhibit *Tb*CLK1 or related factors in parasites that cause other neglected tropical diseases. Importantly, the amino-acid sequences of human and *T. brucei* CLK1s are dissimilar, so *Tb*CLK1 holds promise as a drug target. Further studies should enable the development of inhibitors that preferentially bind the parasite kinase and cure trypanosomiasis without being toxic to humans.
