# Correction: Closing in on a new treatment for sleeping sickness

**DOI:** 10.7554/eLife.01221

**Published:** 2013-07-23

**Authors:** Emily R Derbyshire, Jon Clardy

Derbyshire ER, Clardy J. 2013. Closing in on a new treatment for sleeping sickness. *eLife* **2**:e01042. doi: http://dx.doi.org/10.7554/eLife.01042. Published 09 July 2013

The title of the related research article was incorrect in two locations, next to the accompanying image in the key information box and in the reference list. The correct title of the related research article is: Hypothemycin, a fungal natural product, identifies therapeutic targets in *Trypanosoma brucei*.

The article has been corrected accordingly.

